# Ectasia Risk Assessment Before Laser Vision Correction: The Evolving Role of Corneal Pachymetry

**DOI:** 10.3390/jcm15218560

**Published:** 2026-11-03

**Authors:** Tomás Loureiro, João Heitor Marques, Nuno Campos, Tiago Monteiro

**Affiliations:** 1 Ophthalmology Department, Hospital Garcia de Orta, 2805-267 Almada, Portugal; nunocampos.dr@gmail.com; 2 NOVA Medical School, Faculdade de Ciências Médicas, NMS, FCM, Universidade NOVA de Lisboa, 1169-056 Lisboa, Portugal; 3 Ophthalmology Department, Centro Hospitalar Universitário de Santo António, 4050-342 Porto, Portugal; joaoheitormarques@gmail.com; 4 Ophthalmology Department, Centro Hospitalar Universitário de São João, 4200-319 Porto, Portugal; monteiro.tiago.pt@gmail.com

**Keywords:** laser vision correction, corneal ectasia, thin corneas

## Abstract

**Background/Objectives**: This study aims to review the evolution of preoperative ectasia risk assessment before laser vision correction (LVC), with particular emphasis on the changing role of corneal pachymetry and its interpretation within contemporary multimodal screening strategies. **Methods**: A structured narrative review of the literature was conducted to examine the historical development of ectasia screening, the incorporation of pachymetric parameters into successive risk models, and the available evidence regarding refractive outcomes in constitutionally thin corneas undergoing laser vision correction. **Results**: Preoperative ectasia screening has evolved from reliance on isolated clinical and topographic parameters to comprehensive multimodal assessment integrating corneal tomography, biomechanics, epithelial thickness mapping, and artificial intelligence-based indices. Although corneal pachymetry remains a key component of most contemporary risk models, increasing evidence suggests that constitutionally thin corneas with otherwise normal tomographic and biomechanical profiles can achieve favourable outcomes following surface ablation. However, the available evidence remains limited by retrospective study designs, highly selected populations, and insufficient long-term follow-up. **Conclusions**: Corneal thickness should no longer be interpreted as an isolated indicator of ectasia risk but rather as one component of an integrated assessment of corneal susceptibility. Contemporary preoperative screening should prioritise individualised multimodal evaluation over reliance on isolated pachymetric thresholds. At the same time, further prospective studies are needed to define the long-term safety of surface ablation in constitutionally thin corneas.

## 1. Introduction

Post-refractive surgery corneal ectasia is a progressive, non-inflammatory biomechanical disorder characterized by localized corneal thinning, anterior protrusion, irregular astigmatism, and progressive visual deterioration following laser vision correction (LVC). First described by Seiler et al. in 1998 [1] after laser in situ keratomileusis (LASIK), it rapidly became recognized as one of the most devastating complications of refractive surgery. This is due to its potential to cause irreversible visual impairment and, in advanced cases, the need for corneal transplantation [1,2]. Current evidence suggests that postoperative ectasia results from the interaction between the biomechanical impact of surgery and the intrinsic susceptibility of the cornea. Accordingly, ectasia may develop in eyes with unrecognized ectatic disease, in apparently normal but biomechanically susceptible corneas, or when excessive tissue alteration exceeds the biomechanical reserve of an otherwise healthy cornea [3,4].

The reported incidence (0.02–0.6%) of postoperative ectasia has declined substantially over the past two decades, largely owing to advances in preoperative screening rather than improvements in surgical technique alone [5]. Corneal tomography, biomechanical assessment, epithelial thickness mapping, and integrated diagnostic indices have considerably enhanced the identification of susceptible corneas before surgery. These advances allow many high-risk candidates to be excluded from treatment [4,6,7]. Nevertheless, the biomechanical impact of the intended procedure remains an important determinant of risk. LASIK has consistently been associated with a higher incidence of postoperative ectasia than photorefractive keratectomy (PRK), primarily because flap creation disrupts the anterior stromal lamellae and weakens the biomechanical integrity of the cornea. However, the occurrence of ectasia following PRK and other flapless procedures confirms that intrinsic corneal susceptibility, rather than surgical technique alone, ultimately determines postoperative risk [4,8,9].

Current strategies for ectasia risk assessment have evolved from predictive models derived predominantly from retrospective LASIK cohorts treated during an earlier era of refractive surgery. At that time, preoperative screening relied mainly on anterior corneal topography, corneal thickness, and conventional clinical risk factors. As multimodal imaging has evolved, the ability to identify structurally susceptible corneas has improved substantially. This has allowed many eyes that would previously have undergone surgery to be excluded before treatment. Consequently, the relative contribution of individual risk factors to contemporary screening may no longer reflect that observed in the original derivation cohorts. This issue is particularly relevant for corneal pachymetry, historically regarded as one of the principal determinants of surgical eligibility. Although reduced corneal thickness has consistently been associated with postoperative ectasia in historical studies, increasing evidence suggests that constitutionally thin corneas with otherwise normal tomographic and biomechanical profiles can achieve excellent refractive outcomes. This raises important questions as to whether the historical importance attributed to corneal pachymetry remains justified in the era of contemporary multimodal screening [10].

The aim of this review is to critically appraise the contemporary approach to preoperative ectasia risk assessment before laser vision correction, with particular emphasis on the interpretation of corneal pachymetry within modern multimodal screening strategies. We review the historical evolution of ectasia screening and discuss the evidence supporting the role of corneal thickness in current refractive surgery practice. We also explore whether pachymetric thresholds derived predominantly from historical LASIK cohorts remain applicable in the era of contemporary multimodal assessment and across different refractive procedures.

## 2. Materials and Methods

A structured narrative review was conducted to evaluate the evolving role of corneal pachymetry in preoperative ectasia risk assessment before LVC following the methodological recommendations for narrative biomedical reviews outlined by Gasparyan et al. [11]. A comprehensive literature search was performed in PubMed/MEDLINE from database inception to July 2026. The search strategy combined controlled vocabulary terms, when applicable, and free-text keywords related to corneal ectasia, refractive surgery, corneal thickness, and preoperative screening. Search terms included combinations of “corneal ectasia”, “post-LASIK ectasia”, “post-PRK ectasia”, “laser vision correction”, “LASIK” “PRK”, “KLEX”, “corneal pachymetry”, “central corneal thickness”, “thin cornea”, “ectasia risk assessment”, “corneal topography”, “corneal tomography”, “corneal biomechanics”, “epithelial thickness mapping”, “residual stromal bed”, “percent tissue altered”, “risk score”, and “artificial intelligence”. A targeted PubMed search was also conducted to identify studies evaluating clinical outcomes of photorefractive keratectomy in thin corneas. Search terms included combinations of “photorefractive keratectomy”, “PRK”, “surface ablation”, “transepithelial PRK”, “thin cornea”, “corneal thickness”, and “treatment outcome”. The final refined search retrieved 13 records.

The reference lists of relevant original studies and review articles were also screened to identify additional eligible publications not retrieved through the electronic search.

### 2.1. Study Eligibility and Selection

Original clinical studies, cohort and case–control studies, diagnostic accuracy studies, systematic reviews, meta-analyses, and landmark publications were considered eligible if they addressed the historical evolution of ectasia risk assessment, the role of corneal pachymetry in patient selection, outcomes of refractive surgery in thin corneas, or the development and validation of risk models incorporating pachymetric parameters. Studies evaluating corneal tomography, biomechanics, epithelial thickness mapping, and integrated diagnostic indices were also included when relevant to interpreting corneal thickness within a contemporary multimodal screening strategy. Studies focused exclusively on the diagnosis or management of established keratoconus without direct relevance to refractive surgery screening were excluded. Titles and abstracts were screened for relevance, followed by full-text assessment of potentially eligible articles. Preference was given to studies with higher methodological quality, larger sample sizes, independent validation, and relevance to contemporary ectasia risk assessment. Landmark publications that shaped the historical evolution of preoperative screening were intentionally included irrespective of study design. A total of 30 publications were referenced in the narrative synthesis, encompassing original clinical investigations, diagnostic and methodological studies, systematic reviews, and landmark publications relevant to ectasia risk assessment and corneal pachymetry. Artificial intelligence-assisted tools (ChatGPT (version 6), OpenAI, and Claude (version 5.1), Anthropic) were used to support the identification of potentially relevant publications and the organization of the literature. The relevance, eligibility, and scientific content of the identified studies were independently assessed and verified by the authors.

### 2.2. Evidence Synthesis

Given the heterogeneity of study designs, patient populations, surgical procedures, outcome definitions, and reported endpoints, quantitative meta-analysis was considered inappropriate. The available evidence was therefore synthesized narratively, focusing on the historical evolution of preoperative ectasia screening and the changing role of corneal pachymetry within this process. Attention was given to the incorporation of pachymetric parameters into successive generations of ectasia risk models, the evidence supporting refractive surgery in constitutionally thin corneas with otherwise normal tomographic and biomechanical findings, and the applicability of historical pachymetric thresholds in the era of contemporary multimodal corneal assessment. Evidence derived from LASIK and PRK populations was reviewed separately whenever possible to identify potential procedure-specific differences in the interpretation and clinical relevance of corneal thickness.

## 3. Historical Evolution of Ectasia Screening

The first reports of post-LASIK ectasia highlighted the need for reliable preoperative screening strategies capable of identifying susceptible eyes before refractive surgery. Early screening relied primarily on slit-lamp examination and Placido-based anterior corneal topography, based on the recognition that abnormal corneal morphology represented the strongest predictor of postoperative biomechanical failure. The work of Rabinowitz and colleagues fundamentally transformed this approach by establishing computerized videokeratography as the cornerstone of keratoconus detection. The introduction of quantitative topographic indices—including inferior–superior (I–S) asymmetry, simulated keratometry (K), skewed radial axes (SRAX), and later the Keratonus Percentage Index (KISA)—enabled a more objective identification of both manifest and subclinical keratoconus, substantially improving patient selection compared with subjective topographic interpretation alone [12,13,14]. These developments established the concept that ectasia prevention depended primarily on identifying pre-existing structural abnormalities rather than predicting the biomechanical consequences of surgery itself.

As additional cases of postoperative ectasia continued to emerge despite apparently normal preoperative topography, it became evident that anterior corneal curvature alone could not explain all cases. Clinical observations progressively identified younger age, higher myopic corrections, reduced central corneal thickness (CCT), and lower residual stromal bed (RSB) thickness as additional factors associated with postoperative ectasia. These observations culminated in the development of the Ectasia Risk Score System (ERSS) by Randleman et al. in 2008 [2], representing the first widely adopted multivariable model for preoperative ectasia risk assessment. Based on a retrospective case–control analysis, the ERSS incorporated five variables—topographic pattern, RSB thickness, age, central corneal thickness, and manifest refraction—assigning a weighted score to each according to its estimated contribution to postoperative ectasia risk [2,15].

The ERSS represented a pivotal advance in refractive surgery by recognizing that ectasia risk could not be determined by a single parameter but rather resulted from the interaction of multiple clinical and surgical variables. However, its conclusions should be interpreted within the historical context in which the model was developed. The derivation cohort consisted almost exclusively of LASIK patients treated during an era when preoperative screening relied predominantly on anterior corneal topography and conventional clinical risk factors, before the widespread availability of Scheimpflug tomography, epithelial thickness mapping, in vivo biomechanical assessment, and contemporary integrated diagnostic indices. In addition, many patients underwent LASIK using mechanical microkeratomes and for relatively high refractive corrections, reflecting surgical practices that differ substantially from current standards. These considerations are particularly relevant when interpreting the role attributed to corneal pachymetry. Although reduced CCT was incorporated as one of the five ERSS variables, it was not identified as an independent predictor in the original multivariable regression analysis, raising questions regarding the relative weighting ultimately assigned to pachymetry within the score [2].

Subsequent clinical studies further challenged the concept of reduced corneal thickness as an isolated marker of ectasia susceptibility. Caster et al. reported no cases of postoperative ectasia among more than 1000 LASIK-treated eyes with preoperative CCT between 450 and 500 μm when preoperative topography was normal and surgical planning was appropriate [16]. Similarly, Kymionis et al. demonstrated stable long-term refractive and topographic outcomes following refractive surgery in thin corneas without evidence of progressive ectasia, while Binder concluded that reduced CCT alone was not a consistent independent predictor of postoperative ectasia [9,17]. Collectively, these findings suggested that corneal thickness should not be interpreted as an isolated determinant of surgical eligibility but rather as one component of a broader assessment integrating corneal morphology, biomechanical behaviour, and the anticipated structural impact of surgery.

## 4. The Multimodal Era: From Corneal Thickness to Integrated Ectasia Risk

The introduction of Scheimpflug corneal tomography represented one of the most important advances in preoperative ectasia screening. Unlike Placido-based topography, which is limited to the assessment of anterior corneal curvature, tomography enabled three-dimensional characterization of the cornea, including anterior and posterior elevation, pachymetric distribution, and the spatial relationship between corneal thickness and corneal shape. This represented a fundamental shift in the interpretation of corneal thickness. Rather than relying on CCT or minimum pachymetry as isolated indicators of risk, clinicians could now evaluate the location of the thinnest point and how corneal thickness increased from the center towards the periphery, providing valuable information regarding the structural organization of the cornea [18].

The pioneering work of Ambrósio and colleagues demonstrated that ectatic corneas are characterized not only by reduced thickness but also by an abnormal pachymetric progression profile. This observation led to the development of the Pachymetric Progression Index (PPI) and the Ambrósio Relational Thickness (ART), which relates the thinnest corneal thickness to its progression profile. Both parameters consistently demonstrated superior diagnostic performance compared with minimum pachymetry alone, establishing the concept that the pachymetric profile is considerably more informative than absolute corneal thickness. Consequently, constitutionally thin corneas with physiological pachymetric progression could be distinguished from early ectatic corneas in which focal thinning is accompanied by abnormal thickness progression [18].

Beyond pachymetric profiling, posterior corneal elevation emerged as another key tomographic parameter for the early detection of ectatic disease. Changes at the posterior corneal surface may precede detectable abnormalities in anterior curvature or clinically evident corneal thinning, making posterior elevation one of the earliest tomographic markers of keratoconus. This improved understanding has also led to the reinterpretation of some historical cases of postoperative ectasia following keratorefractive surgery, which may have occurred in eyes with pre-existing subclinical ectatic disease that remained undetected by the preoperative screening techniques routinely available at the time.

These concepts culminated in the development of the Belin–Ambrósio Enhanced Ectasia Display (BAD-D), which integrated anterior elevation, posterior elevation, thinnest pachymetry, pachymetric progression, and relational thickness into a single regression model [19,20]. BAD-D rapidly became one of the most widely adopted tomographic screening tools because of its excellent diagnostic performance for clinical and subclinical ectasia. Nevertheless, BAD-D should be regarded primarily as a diagnostic classifier rather than a true predictor of postoperative ectasia. Moreover, although it represents a major advance over isolated pachymetric assessment, corneal thickness remains represented through multiple highly correlated variables within the algorithm, meaning that pachymetry continues to exert substantial influence on the final score despite its more sophisticated integration [21].

The subsequent incorporation of corneal biomechanics further enhanced ectasia detection. The Corvis Biomechanical Index (CBI) combined dynamic corneal deformation parameters with the horizontal Ambrósio Relational Thickness (ARTh), representing the first clinically applicable integration of structural and functional corneal assessment [6]. This was followed by the Tomographic Biomechanical Index (TBI), which combined Pentacam tomography with Corvis ST biomechanical parameters using machine-learning algorithms and demonstrated superior diagnostic performance compared with either modality alone, particularly in very early or asymmetric ectatic disease [7]. More recently, artificial intelligence-based models, including the Pentacam Random Forest Index (PRFI), BAD-D version 4, and Branched Artificial Intelligence Network (BrAIN), have further improved the detection of subtle ectatic abnormalities through increasingly sophisticated integration of tomographic, biomechanical, and morphological variables [22,23]. Although these models represent the current state of the art in ectasia detection, corneal thickness [24,25] remains embedded within each algorithm, either directly or indirectly, through pachymetric, relational, or biomechanically dependent variables. Within the Corvis ST biomechanical staging model, CCT emerged as the dominant contributor, highlighting that most deformation-derived parameters are substantially dependent on corneal thickness and should therefore be interpreted in the context of pachymetry [26].

In parallel, attention shifted towards estimating not only intrinsic corneal susceptibility but also the biomechanical impact of the intended surgical procedure. The Percent Tissue Altered (PTA) represented a major conceptual advance by expressing the proportion of preoperative corneal thickness affected by flap creation and stromal ablation rather than relying exclusively on RSB thickness [27]. More recently, the Relational Tissue Altered (RTA) index incorporated additional tomographic and structural relationships using machine-learning techniques in an attempt to provide a more individualized estimate of surgery-induced biomechanical impact [28]. Nevertheless, both indices fundamentally depend on preoperative corneal thickness, meaning that thinner corneas inevitably generate greater relative tissue alteration for an equivalent refractive treatment.

An additional consideration is the substantial genetic contribution to corneal thickness and biomechanical properties. Family and twin studies have demonstrated high heritability of central corneal thickness, with estimates ranging from approximately 68% in parent–offspring analyses to 95% in twin populations [24,25]. Corneal hysteresis has also been shown to have a significant hereditary component [29]. Furthermore, genome-wide association studies have identified genetic loci associated with corneal thickness, some of which are also associated with keratoconus susceptibility [30]. These findings suggest that reduced corneal thickness may, in some individuals, represent a constitutionally determined anatomical characteristic rather than an isolated indicator of ectatic disease. Nevertheless, genetic heritability does not establish biomechanical normality or postoperative safety, reinforcing the importance of interpreting pachymetry within a comprehensive multimodal assessment.

Collectively, these developments have transformed preoperative ectasia screening from the interpretation of isolated variables into a comprehensive multimodal assessment integrating corneal morphology, biomechanics, epithelial architecture, and surgical planning. However, multimodal integration should not be interpreted as independence from corneal thickness. Rather, pachymetry remains embedded throughout contemporary risk assessment in multiple forms, including absolute thickness, pachymetric progression, relational thickness indices, biomechanical parameters influenced by thickness, and procedure-related metrics such as PTA and RTA. Consequently, constitutionally thin corneas may contribute simultaneously to several components of the same algorithm, potentially amplifying the apparent influence of pachymetry on the final risk estimate. Whether this reflects true biomechanical susceptibility or repeated mathematical representation of a normal anatomical characteristic remains an important unresolved question [31].

The experience with keratorefractive lenticule extraction (KLEx) further illustrates the limitations of relying on preoperative corneal thickness as an isolated marker of ectasia susceptibility. Although small-incision lenticule extraction (SMILE), the most extensively studied KLEx procedure, has been proposed to offer greater biomechanical stability than LASIK through preservation of the anterior stromal architecture, postoperative ectasia has nevertheless been reported [32]. In a large retrospective series of 6619 eyes undergoing SMILE, Brar et al. identified 10 eyes that developed ectasia, corresponding to an incidence of 0.15%. Notably, these eyes did not generally have thin corneas preoperatively: CCT ranged from 495 to 589 µm, with a mean of 525.3 µm, and 9 of the 10 eyes had a CCT ≥ 500 µm [31]. Eight of the 10 affected eyes were instead classified as borderline preoperatively, whereas only two had normal preoperative topography [31].

These findings reinforce the distinction between corneal thickness and corneal susceptibility to ectasia. In this series, thin preoperative pachymetry was not identified as a risk factor in any of the ectatic eyes; rather, the reported risk factors included younger age, abnormal tomographic or biomechanical indices, higher refractive correction, and low or borderline residual stromal thickness. Thus, although pachymetry remains relevant to surgical planning and to the amount of residual stromal tissue, a conventionally normal preoperative CCT should not be interpreted as evidence of biomechanical normality. The occurrence of ectasia after KLEx in corneas with predominantly normal baseline thickness further supports contemporary screening strategies that integrate pachymetry with corneal morphology, biomechanical assessment, and the anticipated structural impact of surgery.

Interestingly, contemporary refractive surgery practice appears to have evolved more rapidly than the evidence underpinning many currently available risk models. Constitutionally thin corneas with normal tomography, biomechanics, and epithelial thickness profiles are no longer routinely excluded from laser vision correction. Instead, surgical decision-making has progressively shifted towards individualized risk assessment, in which corneal thickness is interpreted within the broader context of complementary structural and functional information rather than as an isolated exclusion criterion. This paradigm is particularly evident in borderline candidates, for whom many surgeons preferentially recommend surface ablation rather than LASIK because of its lower biomechanical impact. However, this approach remains supported largely by clinical judgement and biomechanical rationale rather than robust procedure-specific evidence. Since most available risk models were developed and validated predominantly in historical LASIK cohorts, whether the same pachymetric thresholds—or even the same weighting attributed to pachymetric variables—should be applied to PRK remains uncertain. This discrepancy between contemporary clinical practice and the evidence underpinning current screening algorithms forms one of the central questions addressed in the present review.

## 5. Clinical Outcomes of Surface Ablation in Thin Corneas

Constitutionally thin corneas, conventionally defined as having a preoperative CCT below 500 μm, represent the clinical scenario in which the discrepancy between historical risk models and contemporary refractive surgery practice becomes most evident. Although many refractive surgeons currently favor surface ablation over LASIK in these eyes because of its lower biomechanical impact, the evidence supporting this approach remains relatively limited. Nevertheless, the available literature has consistently reported favorable visual, refractive, and tomographic outcomes following PRK or transepithelial PRK in carefully selected eyes with reduced corneal thickness. The longest available follow-up series, reported by de Benito-Llopis et al., evaluated 75 eyes with a mean preoperative CCT of 481.5 ± 15.7 μm (range, 438–499 μm) undergoing surface ablation and demonstrated stable topographic findings with no cases of postoperative ectasia over a 10-year follow-up, although 40% of eyes required retreatment because of refractive regression [10]. Earlier studies by Hashemi, Kymionis and colleagues similarly reported stable refractive and topographic outcomes following PRK or LASEK in eyes with preoperative CCT generally ranging between approximately 450 and 500 μm, without evidence of ectatic progression during follow-up periods ranging from several months to just over one year [17,33]. More recently, Li et al. evaluated 408 eyes undergoing transepithelial PRK with a postoperative residual stromal thickness below 350 μm and likewise reported no evidence of progressive posterior corneal protrusion or postoperative ectasia after two years of follow-up [34]. Importantly, however, this cohort was selected using contemporary multimodal screening, including Scheimpflug tomography, BAD-D assessment, Corvis ST biomechanical analysis, and epithelial thickness mapping, while eyes with suspicious tomographic findings or CCT below 430 μm were excluded. Similarly, Sachdev et al. reported favorable short-term outcomes in thin corneas treated with either PRK alone or PRK combined with prophylactic corneal cross-linking [35]. However, because the thinnest and tomographically more suspicious corneas preferentially underwent the combined procedure, the study does not permit conclusions regarding the safety of PRK alone in this subgroup. These individual observations were subsequently reinforced by a recent systematic review and meta-analysis including 946 eyes from 10 observational studies, which demonstrated pooled safety and efficacy indices close to 1.0 and reported no documented cases of postoperative ectasia following surface ablation in eyes with a mean preoperative CCT below 500 μm. Collectively, these findings suggest that constitutionally thin corneas with otherwise normal structural characteristics can achieve excellent visual and refractive outcomes following surface ablation.

Despite these reassuring findings, the current evidence remains insufficient to establish the long-term biomechanical safety of surface ablation in thin corneas. Postoperative ectasia is an exceptionally rare event, and the available studies are almost certainly underpowered to detect clinically meaningful differences in its incidence. Most reports are retrospective observational case series without matched control groups, include relatively small sample sizes, employ heterogeneous definitions of thin cornea, and frequently report follow-up limited to one or two years. Even the largest and longest available series included only 75 eyes, while the recent meta-analysis pooled fewer than one thousand eyes across all included studies. Furthermore, many of the earlier studies predated the widespread implementation of contemporary tomographic, epithelial, and biomechanical assessment, whereas more recent investigations evaluated highly selected populations after excluding eyes with even subtle evidence of structural susceptibility. Consequently, the favorable outcomes reported may reflect the effectiveness of increasingly sophisticated multimodal screening rather than demonstrating that reduced pachymetry itself has little or no biomechanical significance. Importantly, most studies were designed to evaluate refractive outcomes rather than ectasia risk, with limited longitudinal assessment of posterior elevation, corneal biomechanics, epithelial remodelling, or other sensitive markers of subclinical biomechanical instability. Therefore, although the available evidence supports the concept that low corneal thickness alone should not be considered an absolute contraindication to surface ablation, it remains insufficient to define universally safe pachymetric thresholds or to demonstrate that constitutionally thin corneas carry the same long-term ectasia risk as corneas of normal thickness. Well-designed prospective studies incorporating standardized definitions of thin cornea, appropriately matched normal-thickness control groups, contemporary multimodal preoperative screening, procedure-specific analyses, and long-term tomographic and biomechanical follow-up are still required to determine the true long-term efficacy and safety of surface ablation in this increasingly common group of patients. Taken together, the available evidence suggests that the question is no longer whether thin corneas can undergo surface ablation, but rather how to identify those thin corneas that are truly biomechanically normal.

## 6. Conclusions

The evolution of preoperative ectasia screening over the past two decades has fundamentally transformed refractive surgery. Advances in corneal tomography, biomechanical assessment, epithelial thickness mapping, and artificial intelligence-based risk models have substantially improved the identification of susceptible corneas, contributing to the progressive decline in postoperative ectasia. Within this evolution, the role of corneal pachymetry has also changed. Historically regarded as one of the principal determinants of surgical eligibility, corneal thickness is now better understood as one component of a complex structural and biomechanical system whose clinical significance depends on its integration with tomographic, biomechanical, epithelial, and surgical variables rather than on absolute pachymetric thresholds alone.

Although contemporary multimodal algorithms represent a major advance over historical screening strategies, corneal thickness remains embedded within most current risk models, either directly or indirectly, through pachymetric progression, relational thickness indices, biomechanical parameters, or estimates of surgery-induced tissue alteration. Consequently, constitutionally thin corneas may accumulate risk across multiple correlated variables, potentially magnifying the apparent contribution of pachymetry to the final risk estimate. This does not diminish the importance of corneal thickness but rather reinforces that it should no longer be interpreted as an isolated surrogate for biomechanical susceptibility.

Current clinical practice has progressively evolved beyond much of the historical evidence from which available risk models were derived. Increasingly, refractive surgeons offer surface ablation to carefully selected patients with constitutionally thin corneas when tomographic, biomechanical, epithelial, and clinical findings are otherwise reassuring. Although the available literature consistently reports favorable visual and refractive outcomes with very few reported cases of postoperative ectasia in these patients, the supporting evidence remains predominantly retrospective, involves highly selected populations, and is limited by heterogeneous inclusion criteria, relatively small sample sizes, and insufficient long-term follow-up. Consequently, the current evidence remains insufficient to define universally safe pachymetric thresholds or to determine whether constitutionally thin corneas undergoing surface ablation carry the same long-term risk of ectasia as corneas of normal thickness.

Ultimately, the challenge in contemporary refractive surgery is no longer identifying the thin cornea but identifying the thin cornea that is truly biomechanically normal. Addressing this question will require well-designed prospective studies that use standardized definitions of thin cornea, contemporary multimodal screening protocols, procedure-specific analyses, appropriately matched control groups, and long-term tomographic and biomechanical follow-up. Such studies will be essential not only to validate the long-term safety of surface ablation in constitutionally thin corneas, but also to refine individualized risk assessment and define the true role of corneal pachymetry in modern refractive surgery. Future advances in ectasia screening are therefore unlikely to arise from new pachymetric thresholds, but rather from more accurate identification of biomechanical susceptibility through truly individualized multimodal assessment.

## Data Availability

No new data were created or analyzed in this article. Data sharing is not applicable.
